# Comparative Structural Modeling Suggests Distinct Signatures of Conformational Plasticity and Surface Physicochemistry in Phytoene Synthase and Dehydrosqualene Synthase

**DOI:** 10.3390/molecules31121995

**Published:** 2026-06-07

**Authors:** Ade Rizqi Ridwan Firdaus, Muhammad Yusuf, Shun Tamaki, Keiichi Mochida, Toto Subroto

**Affiliations:** 1 Center for Sustainable Resource Science, RIKEN, Yokohama 230-0045, Japan; aderizqi.firdaus@riken.jp (A.R.R.F.); m.yusuf@unpad.ac.id (M.Y.); 2 Department of Chemistry, Faculty of Mathematics and Natural Sciences, Universitas Padjadjaran, Sumedang 45363, Indonesia; 3 Research Center for Molecular Biotechnology and Bioinformatics, Universitas Padjadjaran, Bandung 40133, Indonesia; 4 Department of Biological Science, Faculty of Sciences and Engineering, Yasuda Women’s University, Hiroshima 731-0153, Japan; tamaki.shun61@gmail.com; 5 Microalgae Resource Upcycling Research Laboratory, RIKEN, Yokohama 230-0045, Japan; 6 Kihara Institute for Biological Research, Yokohama City University, Yokohama 244-0813, Japan; 7 School of Information and Data Sciences, Nagasaki University, Nagasaki 852-8521, Japan

**Keywords:** phytoene synthase, dehydrosqualene synthase, carotenoid biosynthesis, molecular dynamics

## Abstract

Carotenoids are essential metabolites involved in photosynthesis, cellular protection, pigmentation, and antioxidant activities. Phytoene synthase (PSY/CrtB) utilizes C20 substrates in carotenoid biosynthesis, whereas its structural homolog, dehydrosqualene synthase (CrtM), preferentially accepts C15 substrates. Although previous studies have identified CrtM mutations that expand substrate scope, the molecular basis of substrate discrimination in PSY/CrtB remains poorly understood, largely because of the absence of experimentally determined three-dimensional structures. Here, we integrated comparative sequence analysis, homology modeling, and molecular dynamics (MD) simulations to investigate the structural basis of substrate discrimination in PSY/CrtB. Comparative sequence analysis suggested distinct overall conservation landscapes in PSY/CrtB and CrtM, with 20 highly conserved positions shared between them and clustered around the catalytic core. MD simulations suggest that PSY models exhibit minimal differentiation under cross-ligand conditions, consistent with its greater conformational plasticity. Surface property analysis suggested hydrophobic patches and an amphipathic helix (Helix-13) in PSY that were preferentially conserved in PSY homologs relative to CrtM homologs. Taken together, our analyses suggest that greater conformational plasticity may facilitate the accommodation of C20 substrates in PSY and that its conserved hydrophobic surface architecture may shape its surface physicochemistry. These findings suggest that differences in substrate accommodation between PSY/CrtB and CrtM may reflect coordinated variation in conformational dynamics, pocket hydrophobicity, and surface architecture, rather than substantial alterations to the conserved catalytic core.

## 1. Introduction

Carotenoids are a diverse class of isoprenoid compounds synthesized by a wide range of organisms, including bacteria, algae, and plants, with essential roles in pigmentation, photoprotection, and signaling [1,2,3]. Beyond their biological roles, carotenoids are prominent functional ingredients in the food industry, utilized as natural colorants and bioactive micronutrients with widely reported antioxidant and health-promoting properties [4,5,6]. Carotenoid biosynthesis begins with the C5 isoprenoid precursors isopentenyl diphosphate (IPP) and dimethylallyl diphosphate (DMAPP), which are synthesized via the mevalonate (MVA) or methylerythritol phosphate (MEP) pathway [7,8,9]. The MVA pathway predominates in eukaryotes and archaea, whereas the MEP pathway predominates in plant plastids and eubacteria [10]. Although most organisms rely on only one of these pathways, plants and *Streptomyces* species are notable exceptions that utilize both for isoprenoid biosynthesis [11]. In plants, these two pathways are partitioned into distinct metabolic outputs, with most carotenoids originating from the plastidial MEP pathway [12].

Carotenoid biosynthesis is commonly classified by carbon backbone length, with most pathways producing either C30 or C40 carotenoids [13]. During C40 carotenoid biosynthesis, geranylgeranyl diphosphate synthase catalyzes the sequential addition of three IPP molecules to one DMAPP molecule to generate geranylgeranyl diphosphate (GGPP, C20) [14]. Phytoene synthase (PSY in plants and CrtB in bacteria) catalyzes the head-to-head condensation of two GGPP molecules to form phytoene, the first committed intermediate in C40 carotenoid biosynthesis [15]. Since PSY typically serves as the rate-limiting enzyme in the production of C40 carotenoids, such as β-carotene and lycopene, it is a primary target for metabolic engineering and biofortification strategies aimed at increasing the yield of these high-value health-promoting compounds in food crops and microbial cell factories [15]. In C30 carotenoid biosynthesis, farnesyl diphosphate synthase catalyzes the IPP–DMAPP condensation to generate farnesyl diphosphate (FPP, C15), and dehydrosqualene synthase (CrtM) catalyzes the head-to-head condensation of two FPP molecules to form dehydrosqualene [16].

The structurally homologous enzymes PSY/CrtB and CrtM have been extensively studied to determine how mutational changes alter substrate selectivity for prenyl diphosphates of different chain lengths and to define the amino acid and structural determinants of that specificity [16,17]. CrtM exhibits remarkable functional plasticity: mutations at residue F26 (e.g., F26A, F26S, F26L) enable CrtM to accommodate two molecules of GGPP (C20) and synthesize the C40 carotenoid phytoene, and additional mutations in these F26 variants further extend the enzyme’s capacity to produce longer-chain carotenes, such as C50 and potentially C60 compounds [18,19,20]. By contrast, PSY/CrtB appears far less adaptable; despite extensive mutagenesis and screening efforts, no CrtB mutants with C30-carotenoid synthase activity have been reported [16,17]. This contrast highlights the limited understanding of why PSY/CrtB appears to exclude C15 substrates. The absence of an experimentally determined three-dimensional structure for CrtB further constrains mechanistic insight into this stringent substrate specificity.

To address this gap, we integrated comparative sequence analysis, homology modeling, and molecular dynamics (MD) simulations to characterize the structural basis and mechanistic differences between PSY/CrtB and CrtM. Specifically, we aimed to (1) map the conservation landscapes of PSY/CrtB and CrtM, (2) generate and assess a structural model of PSY, (3) characterize ligand-dependent conformational dynamics using MD simulations, and (4) identify surface physicochemical features that may distinguish PSY from CrtM.

## 2. Results

### 2.1. Comparative Evolutionary Context of PSY/CrtB and CrtM

**Figure 1:**
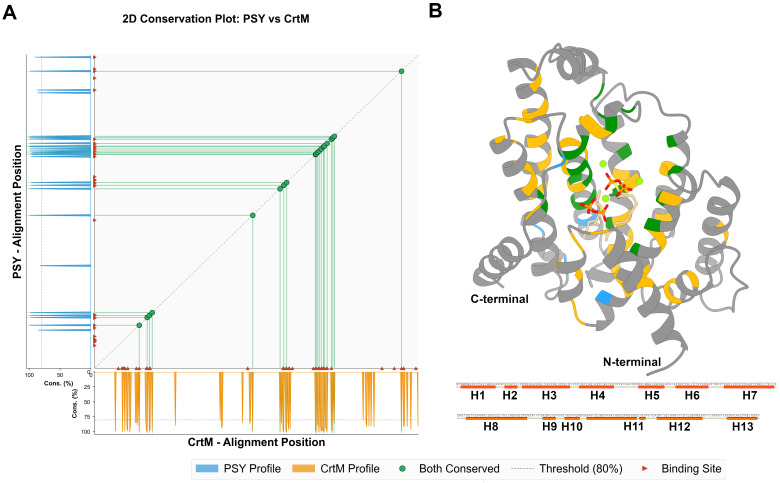
Comparative conservation analysis of CrtB/PSY and CrtM proteins. (**A**) Conservation profiles for PSY/CrtB (blue) and CrtM (orange) derived from independent sequence alignments. Bars indicate residue conservation at each alignment position, and the dashed lines mark the 80% threshold. Shared conserved positions are connected by green lines and circles. Overall, CrtM showed more highly conserved positions (*n* = 63) than PSY/CrtB (*n* = 26), with 20 residues conserved in both groups. Substrate-binding site residues are marked by red triangles. (**B**) Conserved residues mapped onto the three-dimensional structure. The protein is shown in gray ribbon representation, with the ligands shown as sticks and the Mg^2+^ ions displayed as neon green balls. Residues conserved in both groups are shown in green, whereas residues uniquely conserved in CrtM and PSY/CrtB are shown in orange and blue, respectively. The red line shows the secondary structure map, with H1 to H13 indicating helix numbers.

To characterize the broad evolutionary context of CrtM and PSY/CrtB, we collected putative members of both enzyme families from the KEGG database and analyzed their conservation patterns. Comparison of the two sequence alignments highlighted a discrete set of positions that were highly conserved within both families, as shown in the diagonal conservation plot (Figure 1A). Overall, 20 positions were conserved in both datasets, whereas family-specific conservation was more pronounced in CrtM; 43 positions were specifically conserved in CrtM, compared to only six in PSY/CrtB (Figure 1A). To examine the spatial distribution of these residues, we mapped them onto the CrtM structure (PDB: 3W7F) and found that they cluster around the central ligand-binding pocket and core α-helices (Figure 1B). These comparisons suggest that the two enzyme families retain a conserved ligand-binding region.

To assess the conformational variability of the conserved helical framework, we compared the available CrtM structures representing the apo, holo, and inhibitor-bound states (apo: 2ZCO, holo: 3W7F, and 5IYS, inhibitor-bound: 2ZCQ) using Protein Contacts Atlas [21]. Interhelical contact patterns differed across these states, with the apo state showing a denser network of helix–helix interactions than the holo forms (Figure S1). These differences were concentrated in the helical scaffold surrounding the catalytic cavity. Detailed contact mapping further suggested that interactions linking Helix-2 and Helix-3 to the surrounding helical core were preserved in ligand-bound states but weakened or absent in the apo structure. Together, these observations suggest that the conserved region surrounding the catalytic pocket adopts state-dependent conformational arrangements in CrtM, motivating the structure-based analysis of the corresponding region in PSY.

**Figure 2:**
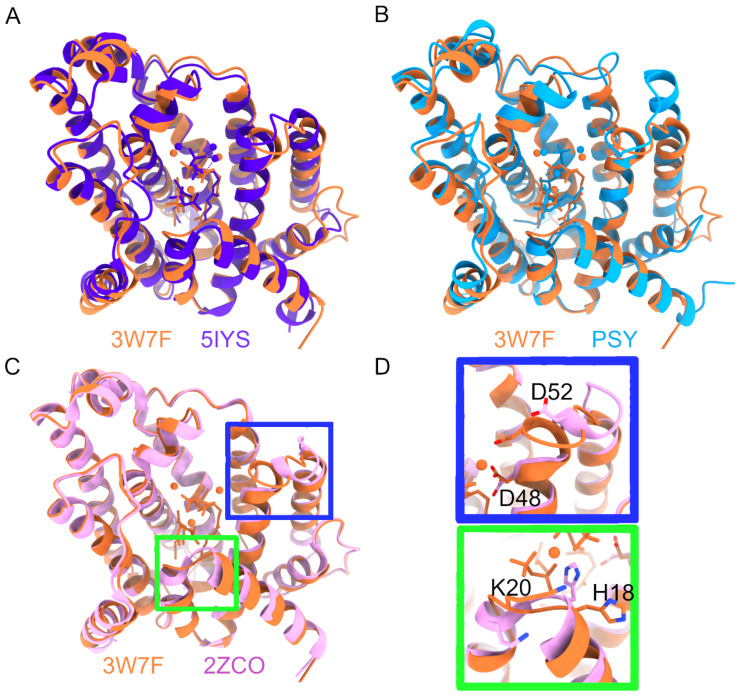
Structural comparison of CrtM crystal structures and the PSY model. (**A**) Overlay of the holo CrtM structures 3W7F (orange) and 5IYS (purple), highlighting alternative Mg^2+^ coordination patterns in the active site. 3W7F shows a more symmetric metal arrangement, whereas 5IYS displays a shifted configuration with two ions associated with the Helix-3 DXXXD motif and one with the Helix-8 motif. (**B**) Superposition of the experimental CrtM structure 3W7F (orange) and the OpenFold3 model of PSY (cyan). The comparison shows broad agreement in overall fold and cavity architecture between the experimental and predicted structures. (**C**) Overlay of the holo CrtM structure 3W7F (orange) and the apo structure 2ZCO (pink), showing that the global helical framework is largely preserved between the two states. (**D**) Close-up views of the boxed regions in panel C. The upper inset shows that D48 remains in similar positions between the apo and holo forms, while D52 is largely in an open conformation. The lower inset shows H18 positioned at the loop between helix-1 and helix-2, which also displays a distinct conformation between the apo and holo forms, similar to D52.

The available CrtM structures further suggest that the active-site environment is not represented by a single rigid configuration. Comparison of the two holo structures, 3W7F and 5IYS, suggested distinct Mg^2+^ coordination arrangements within the catalytic cavity, consistent with multiple substrate- and cofactor-associated structural states (Figure 2A). A comparison of these structures with the apo form suggested that although the overall helical bundle remained largely superimposable, local features of the catalytic environment varied across states (Figure 2C). In particular, the Asp-rich metal-binding residues D48 and D52, as well as H18, which helps stabilize the substrate diphosphate group, adopt different orientations in the holo structures [22]. Since no experimental structure is available for PSY, we analyzed an OpenFold3-generated PSY model to assess whether PSY preserves the corresponding structural framework. The model closely recapitulated the overall helical scaffold and spatial organization of the pyrophosphate–magnesium binding geometry observed in the CrtM holo structure (Figure 2B), providing a structural framework for subsequent comparative molecular dynamics analysis.

### 2.2. Distinct Ligand-Dependent Conformational Behaviors in PSY and CrtM

**Figure 3:**
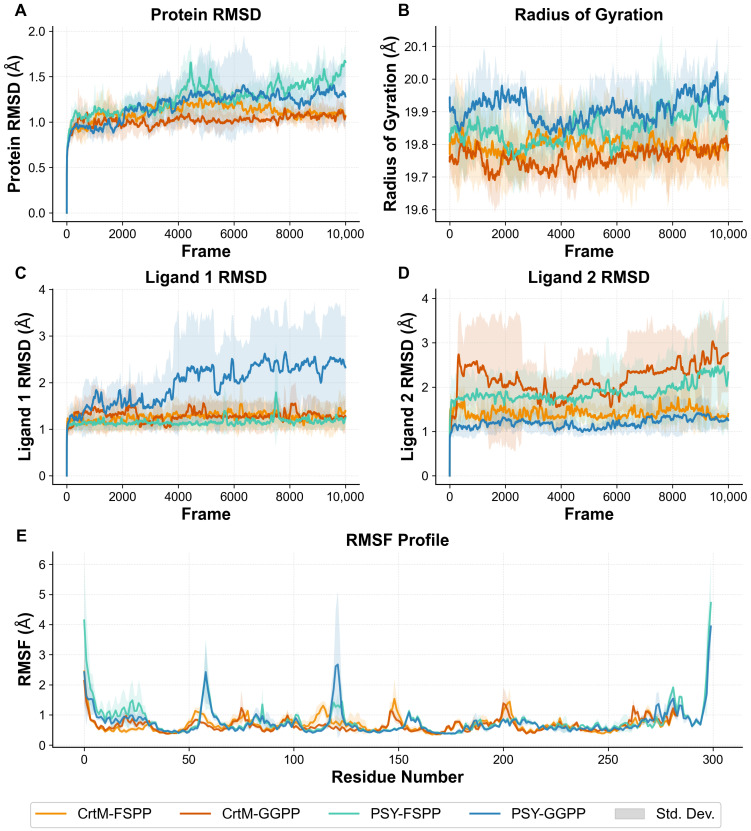
Molecular dynamics simulations of CrtM and PSY under native and substrate-swapped ligand conditions. (**A**) Protein backbone RMSD over the 200 ns trajectories for CrtM and PSY in complex with either the native or swapped substrate (FSPP or GGPP). (**B**) Radius of gyration (Rg) as a function of simulation time, showing differences in overall compactness among the simulated complexes. (**C**,**D**) Ligand RMSD for ligand 1 (Substrate 1) and ligand 2 (Substrate 2), respectively, monitored throughout the simulations to assess substrate stability within the binding cavity. (**E**) Residue-wise RMSF profiles for all systems. Shaded regions indicate the standard deviation across replicates.

To evaluate the structural stability and dynamic behavior of CrtM and PSY in the presence of FSPP and GGPP, we performed 400 ns all-atom molecular dynamics (MD) simulations for each enzyme–ligand combination and used the final 200 ns of each trajectory for subsequent analyses. Our convergence analysis indicated that the trajectories reached a plateau after time windows of 50–75 ns (Figure S2). We also observed that the backbone RMSD fluctuated between 1.0 and 2.0 Å (Figure 3). Despite this overall stability, PSY-based systems exhibited higher RMSD values than CrtM systems. RMSD distributions likewise suggested that PSY-based systems sampled a broader range and showed higher median values than the more compact CrtM distributions. Residue-level RMSF profiles suggested localized flexible regions: while the core residues (positions 50–250) remained relatively stable across all systems (<2 Å), elevated fluctuations were observed at the N-terminus and in specific loop regions, particularly around residues 60 and 290. The radius of gyration (Rg) remained consistent throughout the trajectories (approximately 19.6–20.4 Å), supporting the observation that the global fold and overall compactness of the proteins were maintained during the simulations. Ligand RMSD analysis suggested that ligand 1 remained stable within the binding pocket in most replicates, although several PSY-GGPP replicates exhibited greater substrate mobility (Figure 3C). Together, these findings suggest that both enzyme systems maintained their overall structural integrity during the simulations, whereas the PSY systems sampled a broader conformational space than CrtM.

**Figure 4:**
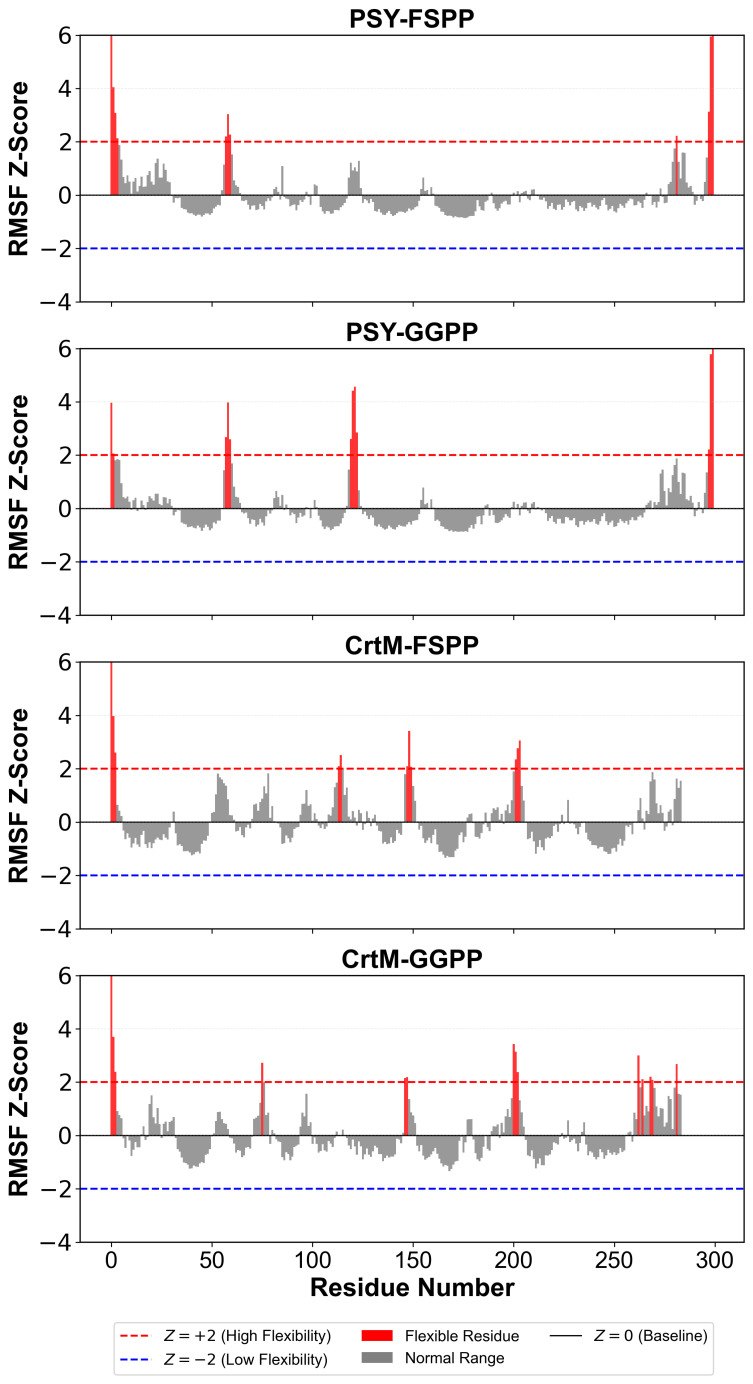
Residue-level flexibility profiles based on RMSF Z-scores. Residue-wise RMSF Z-score profiles are shown for PSY–FSPP, PSY–GGPP, CrtM–FSPP, and CrtM–GGPP (top to bottom). The *y*-axis represents the RMSF Z-score of each residue relative to the mean fluctuation within each system. Residues with Z-scores above +2 are highlighted in red, indicating unusually high flexibility, whereas residues within the normal range are shown in gray. Dashed horizontal lines denote the reference thresholds for elevated flexibility (Z = +2) and reduced flexibility (Z = −2). All panels are aligned by residue number to facilitate direct comparison of flexibility hotspots across protein scaffolds and ligand-binding states.

**Figure 5:**
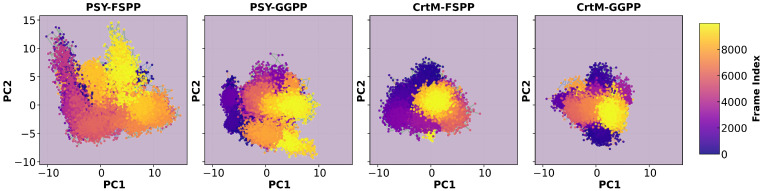
Principal component analysis (PCA) of CrtM and PSY bound to native and swapped prenyl diphosphate substrates. The conformational spaces sampled by PSY–FSPP, PSY–GGPP, CrtM–FSPP, and CrtM–GGPP are shown from left to right as projections onto the first two principal components (PC1 and PC2). Each point corresponds to one simulation frame and is colored by frame index, from early (purple) to late (yellow) stages of the trajectory.

To further define these ligand-dependent differences, we compared residue-level flexibility and global conformational sampling across systems. The RMSF values were converted to Z-scores for each system to enable comparison of relative flexibility profiles (Figure 4). The residue-level flexibility patterns differed markedly between PSY and CrtM. PSY-FSPP and PSY-GGPP shared a prominent flexible cluster around residue 60, whereas PSY-GGPP exhibited a distinct flexibility spike near residue 120, which was absent in the FSPP-bound state. In contrast, CrtM displayed ligand-dependent flexibility peaks at different positions: residue 76 in the FSPP-bound state, and residue 114 in the GGPP-bound state. To compare the breadth of conformational sampling across systems, we conducted PCA of the Cα trajectories (Figure 5). The PSY-FSPP system sampled the broadest conformational space, followed by the PSY-GGPP system, suggesting that PSY may be less conformationally constrained than CrtM. Although PSY-FSPP occupied a larger PCA envelope than PSY-GGPP, the higher Rg values (Table S1) and distinct flexibility peaks of PSY-GGPP suggested that these two metrics capture complementary features of conformational dynamics. In contrast, CrtM displayed a consistently narrow sampling range across both ligand states, suggesting a preorganized architecture. Together, these analyses suggest that ligand-dependent differences between PSY and CrtM are reflected in both the localized flexibility patterns and global conformational sampling.

### 2.3. Distinct Pocket Composition of PSY and CrtM

To investigate the binding pocket features that may underlie the ligand-dependent dynamics described above, we compared the composition of the substrate-binding cavities in CrtM (PDB IDs: 3W7F and 5IYS) and PSY (EgCrtB). The binding cavity was defined as residues located within 5 Å of the substrates and Mg^2+^-binding sites and was partitioned into a catalytic side and a hydrophobic cavity side (Figure S3). The residue compositions of these binding cavities were highly similar, with an overall similarity ranging from 83.8% to 89.2% (Figure S3C). Most conserved positions were concentrated on the catalytic side, including residues such as Y129, Q165, and N168, which were previously implicated in catalysis and Mg^2+^ coordination in CrtM [23]. In contrast, non-identical residues were primarily distributed on the hydrophobic side, including sites corresponding to F26 and F233, which have been associated with substrate-size accommodation and catalytic efficiency in earlier studies [16,18,19]. These findings suggested that PSY and CrtM share a conserved catalytic core, whereas their differences are preferentially localized to the surrounding hydrophobic regions of the pocket.

**Figure 6:**
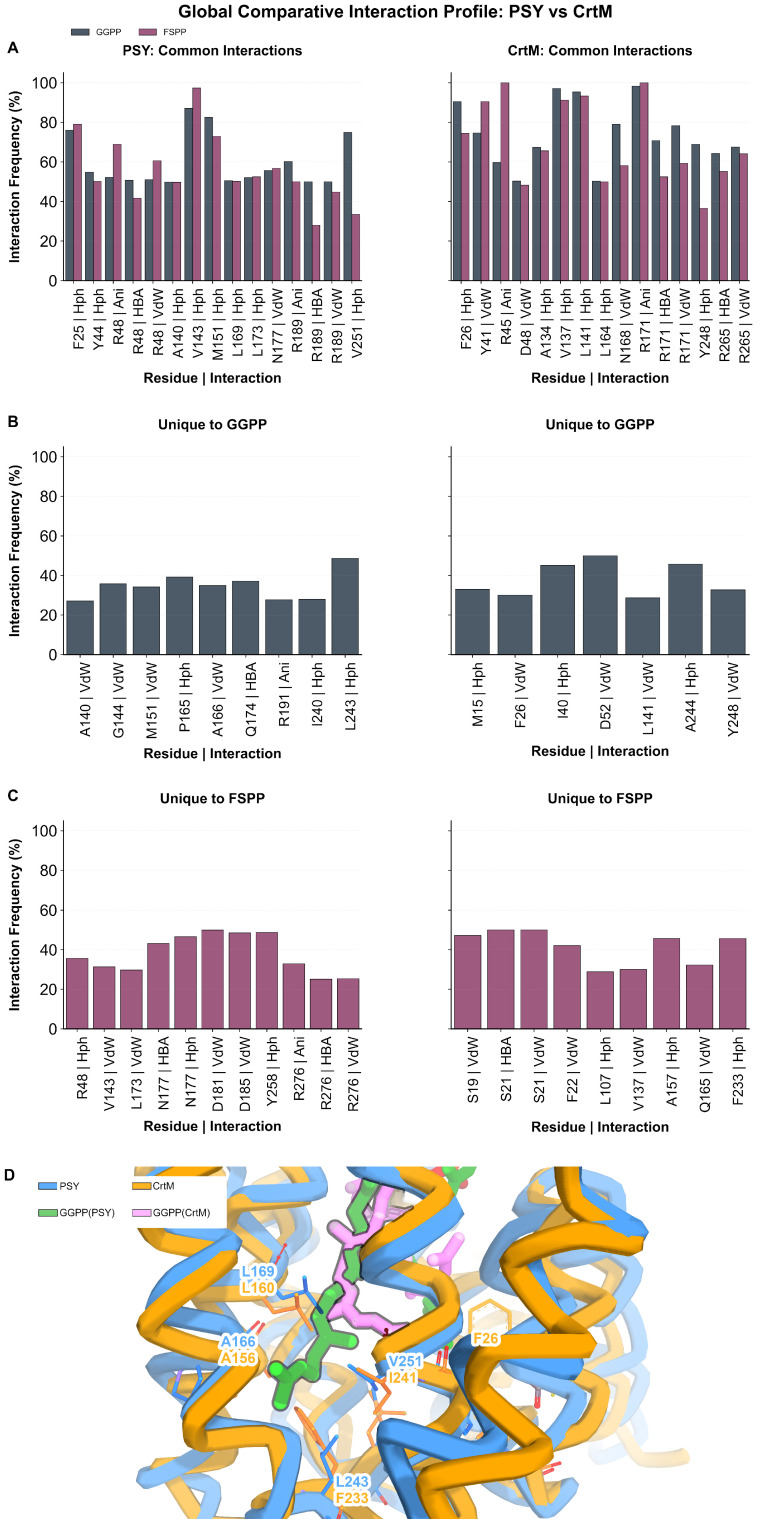
Protein–ligand interaction fingerprints for PSY and CrtM. (**A**) Bar plots summarize the frequencies of specific protein–ligand interactions observed during the MD simulations for PSY (left column) and CrtM (right column). Interactions are classified as common to both ligands, (**B**) unique to GGPP, or (**C**) unique to FSPP. The *x*-axis labels indicate the residue and interaction type, and the *y*-axis shows the percentage of simulation frames in which each interaction was present. GGPP and FSPP are shown in gray and magenta, respectively. (**D**) Structural visualization of GGPP interactions in PSY and CrtM systems after the simulations. The PSY system is represented by a blue ribbon with green GGPP, while the CrtM system is represented by an orange ribbon with pink GGPP.

We examined whether these pocket differences were reflected in distinct ligand-interaction patterns by comparing residue–ligand interaction-frequency fingerprints for PSY and CrtM (Figure 6). Both enzymes retain a conserved diphosphate-anchoring core but differ markedly in their hydrophobic interactions. In CrtM, three arginine residues (R45, R171, and R265) form high-frequency ionic and hydrogen bonds with the diphosphate group, whereas PSY showed a reduced set of anchoring residues (R180 and R189). PSY R48, corresponding to CrtM R45, also contributed to van der Waals and hydrogen bond contacts with both substrates (Figure 6A). However, in the FSPP-bound state, this residue additionally engaged in hydrophobic interactions (Figure 6C,D). Notable differences were observed in the hydrophobic regions of the pocket.

These differences were especially pronounced in the hydrophobic pocket region. In PSY, GGPP binding recruited a dense network of hydrophobic contacts involving L243, M18, I240, and A22, whereas these contacts were largely absent in the FSPP-bound state. In contrast, CrtM appeared to stabilize its native FSPP substrate through prominent polar interactions with S19 and S21; these contacts are reduced upon GGPP binding. Together, these findings suggest that, although the catalytic core is conserved, PSY and CrtM differ substantially in their interaction patterns within the surrounding hydrophobic pocket environment.

### 2.4. A Conserved Hydrophobic Surface Feature in PSY/CrtB

**Figure 7:**
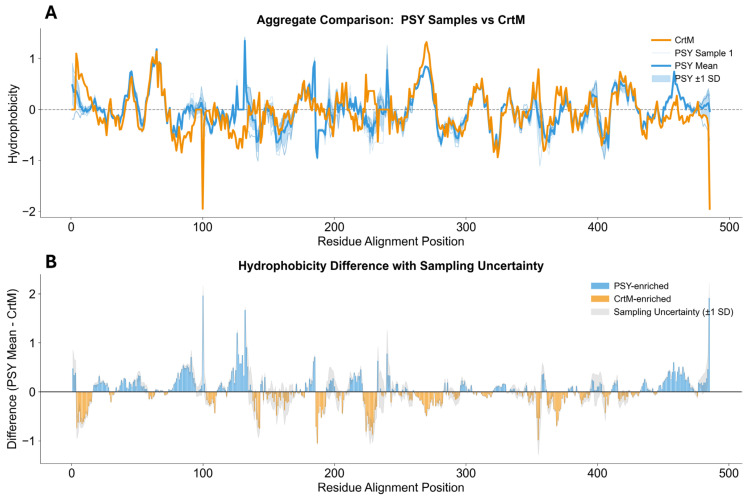
Comparative hydrophobicity profiles of PSY and CrtM. (**A**) Aggregate hydrophobicity profiles based on the Cowan–Whittaker scale (HPLC, pH 7.5). The orange line represents CrtM, whereas the blue line and shaded blue region represent the mean hydrophobicity and its standard deviation of PSY across five samples. (**B**) Difference plot showing hydrophobicity differences between PSY and CrtM (PSY mean − CrtM). Blue bars indicate positions at which PSY is more hydrophobic than CrtM, whereas orange bars indicate positions at which CrtM is more hydrophobic. Gray shading represents sampling uncertainty.

To identify the physicochemical features potentially relevant to substrate recognition, we compared the hydrophobicity profiles of PSY and CrtM using the Cowan–Whittaker scale [24] (Figure 7). PSY displayed higher hydrophobicity than CrtM at positions 81–90 (V53–I57), 110–120 (Q58–Q68), and 460–485 (K270–I287). The sampling uncertainty (gray shaded area) remained low across most positions, suggesting that these differences were robust across the sampled PSY sequences.

**Figure 8:**
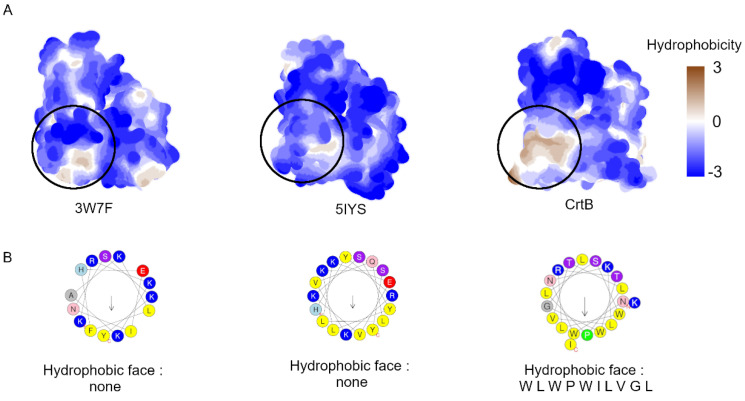
Surface hydrophobicity and helical wheel analysis of CrtM and CrtB. (**A**) Molecular surface representations of 3W7F, 5IYS, and the modeled CrtB, colored by surface hydrophobicity (blue, more polar; brown, more hydrophobic). Circled regions indicate the corresponding surface area compared across the three structures and highlight the distinct hydrophobic patch present in CrtB but absent from the two CrtM structures. (**B**) Helical wheel projections of the corresponding α-helical segments from HeliQuest [25]. The segments from 3W7F and 5IYS do not show a clear hydrophobic face, whereas the PSY segment displays a clustered hydrophobic face composed of W, L, W, P, W, I, L, V, G, and L. The arrow at the center of each helical wheel shows the direction and magnitude of the hydrophobic moment, pointing toward the hydrophobic face of the helix, while the residue colors indicate their physicochemical properties. Yellow represents non-polar residues, other colors represent polar residues, and proline, alanine, and glycine are marked as special residues.

We investigated whether these sequence-level hydrophobicity differences corresponded to structural features. The surface hydrophobic properties of PSY were compared with those of the CrtM structures 3W7F and 5IYS (Figure 8). Although 3W7F and 5IYS displayed predominantly polar surface distributions, PSY contained distinct solvent-exposed hydrophobic patches localized to a C-terminal helix (Helix-13). Helical wheel analysis further distinguished these proteins: the corresponding helices in 3W7F and 5IYS lacked a clearly defined hydrophobic face, whereas the PSY helix displayed a pronounced amphipathic pattern with a continuous hydrophobic face composed of the residues W-L-W-P-W-I-L-V-G-L (mean hydrophobic moment ⟨μH⟩ = 0.49; mean hydrophobicity ⟨H⟩ = 0.76). These observations suggested that the localized hydrophobicity differences identified in Figure 7 corresponded to a discrete surface feature in PSY.

**Figure 9:**
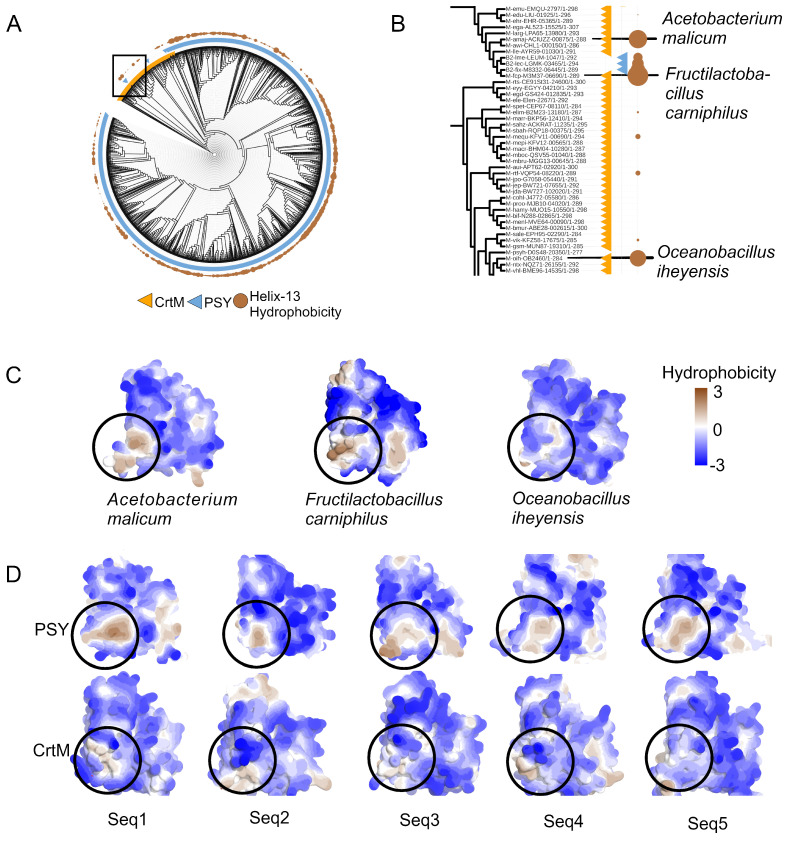
Phylogenetic distribution and structural comparison of Helix-13 hydrophobicity in CrtM and PSY homologs. (**A**) Circular phylogenetic tree of CrtM and PSY homologs. The outer ring distinguishes CrtM (orange), and PSY (blue) clades, whereas brown circle symbols and size indicate average Helix-13 hydrophobicity values based on the Cowan–Whittaker scale (HPLC, pH 7.5). (**B**) Enlarged view of a CrtM-related subclade highlighting the distribution in Helix-13 hydrophobicity among representative homologs. (**C**) Surface hydrophobicity models of three representative species from panel B, colored by hydrophobicity (brown, more hydrophobic; blue, more polar). Circled regions indicate the Helix-13 area. (**D**) Surface hydrophobicity comparison of sequences randomly sampled from the PSY clade (top row) and CrtM clade (bottom row).

We further examined whether this hydrophobic surface feature was conserved across homologs or restricted to the modeled PSY structure. The hydrophobicity of Helix-13 was mapped across a comprehensive phylogenetic tree of CrtM and PSY homologs (Figure 9). This analysis suggested a dichotomy; the PSY clade (blue ring) was consistently associated with high hydrophobicity scores in this region, whereas the CrtM clade (orange ring) showed lower hydrophobicity. Examination of representative clades (Figure 9B) and their corresponding structural models (Figure 9C) showed that the variation in the calculated hydrophobicity was reflected in the surface properties. For example, *Fructilactobacillus carniphilus*, which displayed a relatively high score within a variable clade, exhibited a distinct hydrophobic patch, whereas its lower-scoring relatives, such as *Acetobacterium malicum* and *Oceanobacillus iheyensis*, did not. Random sampling of sequences from major PSY and CrtM clusters (Figure 9D) further supported the observation that PSY orthologs consistently retained this hydrophobic surface feature, whereas CrtM orthologs generally did not. Together, these observations suggest that the Helix-13 hydrophobic surface feature is broadly conserved within PSY homologs but not within CrtM homologs.

## 3. Discussion

Despite their distinct substrate preferences, PSY/CrtB and CrtM share a conserved catalytic scaffold. Our comparative sequence analysis and structural modeling suggested that the PSY model recapitulated the pyrophosphate-binding geometry observed in the CrtM holo structure (Figure 1 and Figure 2). This shared core architecture is consistent with the results of previous mutational studies showing that substrate accommodation in CrtM can be altered by changes in specific binding-pocket residues [18]. Together, these observations suggest that substrate specificity in this enzyme family, particularly in PSY, may be driven by peripheral conformational determinants and physicochemical features.

Our MD analyses suggest that the putative substrate discrimination in PSY may depend not only on static pocket architecture but also on conformational dynamics. All CrtM systems remained globally stable, whereas the PSY-based systems exhibited broader Rg and RMSD distributions, suggesting greater structural heterogeneity despite maintenance of the overall fold (Figure 3, Table S1). This trend is consistent with the broader conformational sampling observed for PSY in principal component space (Figure 5), supporting the view that the PSY scaffold explores a wider dynamic landscape than CrtM. At the residue level, this behavior was reflected in distinct flexibility profiles (Figure 4). Both FSPP- and GGPP-bound PSY shared prominent mobile regions near residue 60, corresponding to loop regions of the enzyme. Flexibility around residue 60 mapped to the short loop connecting helices 3 and 4, which flank the catalytic DXXXD motif and may undergo conformational adjustments during substrate binding. The GGPP-bound state displayed an additional flexibility feature near residue 120, which was absent in the FSPP-bound state. The region around residue 120 corresponded to an extended loop between helices 6 and 7. Notably, these two loop regions were also highlighted as flexible segments in the CrtM crystal structure based on their elevated B-factor values (Figure S4). Although this GGPP-specific mobility peak distinguished the two ligand-bound states, the overall RMSF trend profiles of PSY-FSPP and PSY-GGPP were broadly similar, suggesting that the PSY scaffold could structurally accommodate both FSPP and GGPP without major rearrangements. In contrast, CrtM flexibility profiles differed more substantially between the FSPP- and GGPP-bound states, despite its more compact RMSD distributions. This observation suggests that CrtM, although globally more rigid, may be less tolerant of non-native substrates at the local structural level. Notably, these computational observations are consistent with the experimental finding that PSY displays no significant catalytic activity toward C15 substrates (FPP) [16], implying that structural accommodation alone is insufficient for productive catalysis and that additional factors, such as precise substrate positioning in the catalytic site, are likely required.

The ligand-dependent conformational dynamics observed in PSY were also reflected in distinct binding-pocket interaction patterns. The catalytic side of the pocket is conserved between PSY and CrtM, whereas the hydrophobic side shows greater sequence divergence (Figure S2), consistent with earlier mutational studies demonstrating that F26 and F233 are key determinants of substrate fit and specificity by sterically restricting access to larger substrates [17,18,19,20,26]. These structural differences were captured by interaction fingerprinting, which showed that CrtM relied more heavily on polar interactions, whereas GGPP binding in PSY was associated with a denser hydrophobic contact network (Figure 6). The absence of steric barriers equivalent to those of F26 and F233 in PSY may permit more extensive hydrophobic interactions in the GGPP-bound (C20) state (Figure 6D). However, this alone does not explain PSY’s limited activity toward C15 substrates, suggesting that substrate discrimination involves factors beyond simple steric complementarity, including the specific geometry and dynamics of diphosphate anchoring required for catalytic activation.

The differences between PSY and CrtM may extend beyond the catalytic site. Our analysis identified a distinct hydrophobic patch on Helix-13 of the PSY surface (Figure 8 and Figure 9). The phylogenetic conservation of this patch across the PSY clade suggests that it represents a conserved surface feature linked to PSY-specific functions (Figure 9). Given the amphiphilic character of GGPP [15], this hydrophobic surface may help localize PSY at the membrane interface where substrate access, folding stability, and catalytic activation can be coordinated. Together, these observations suggest that PSY specialization may extend beyond pocket-level substrate discrimination to encompass adaptation to a membrane-associated functional environment not present in the soluble CrtM scaffold.

In summary, our analyses suggest that substrate specificity in PSY/CrtB and CrtM may be shaped less by major alterations to the catalytic core than by coordinated evolution of the surrounding conformational landscape and surface architecture. Although experimentally determined PSY structures in both the apo and holo states will be required to further assess these mechanistic interpretations, the integrative perspective developed here provides a useful framework for rational engineering of carotenoid synthases that incorporate conformational dynamics and hydrophobic surface architecture.

This study has several limitations. First, the PSY structural model was generated using OpenFold3 and could not be benchmarked against an experimentally determined structure. Although the model exhibited good pLDDT and PDE scores (Figure S5) and was in good agreement with the CrtM crystal structure in the conserved core (Figure 2B), its accuracy in surface-exposed loop regions remains uncertain. Second, only one PSY representative (*E. gracilis*) was analyzed and subjected to MD simulations; therefore, the generalizability of the simulated dynamic features across the PSY family is unclear. Third, the 200 ns simulation timescale, which is adequate for capturing local fluctuations, PCA-level sampling, and interaction fingerprints, may not fully capture slow conformational transitions, such as large-amplitude hinge motions. Future progress will depend on the experimental determination of PSY/CrtB structures in both the apo- and substrate-bound states, ideally complemented by hydrogen–deuterium exchange mass spectrometry to directly probe conformational dynamics. At the computational level, the identification of Helix-13 motivates membrane-embedded coarse-grained and all-atom simulations to directly test whether this amphipathic surface mediates PSY–membrane interactions and influences substrate accessibility.

## 4. Materials and Methods

### 4.1. Sequence Analysis

Protein sequences of phytoene synthase (PSY/CrtB) and dehydrosqualene synthase (CrtM) were retrieved from the Kyoto Encyclopedia of Genes and Genomes (KEGG) database [27] using the FASTA protein protocol. In total, 5165 PSY/CrtB sequences and 394 CrtM sequences were collected. Class-specific sequence motifs characteristic of each enzyme class were identified using Uclust (v1.0) [28], JalView (v2.11.5.1 [29], Biopython (v1.84) [30], TrimAl (v1.5.rev1) [31], and the Expasy ProtScale server [32]. To refine the dataset, Uclust removed redundant sequences at a 95% sequence identity threshold, after which TrimAl filtered the alignment by excluding highly divergent sequences at a 50% residue threshold.

Multiple sequence alignments (MSAs) were generated using Clustal Omega [33], and sequence conservation across the aligned families was quantified to detect functionally important residues. Phylogenetic trees were constructed using IQ-TREE 2 [34] under the Q.PFAM+R8 model. Hydrophobicity profiles were assessed using the Cowan–Whittaker scale, which reflects hydrophobicity indices at pH 7.5 determined by high-performance liquid chromatography (HPLC) [24].

### 4.2. Protein Structures

Since no experimentally determined structure was available for PSY, *Euglena gracilis* CrtB (accession: BAU24764.1) [35] was selected as a representative PSY sequence and modeled in complex with GGPP using OpenFold3 [36]. The OpenFold3 model was evaluated using predicted local distance difference test (pLDDT) scores and Predicted Distance Error (PDE). CrtM crystal structures were retrieved from the RCSB Protein Data Bank (PDB) [37] under the following accession codes: 3W7F, 2ZCO, 2ZCQ [22], and 5IYS.

### 4.3. Molecular Dynamics Simulations

#### 4.3.1. Receptor Preparation

The preparation was conducted in two stages. First, each protein PDB file was cleaned by removing all hydrogen atoms using the pdb4amber program provided by AmberTools23 [38]. Second, the protein structures were protonated based on their steric environment, and pKa values were predicted using the PDB2PQR web server [39].

#### 4.3.2. Ligand Preparation

The ligand coordinates were extracted from the respective complex structures. The ligands were protonated using the pKaNET Cloud (https://github.com/nyelidl/pKaNET_Cloud (accessed on 21 January 2026) to generate the protonated state at pH 7.4 and the fully deprotonated state. The resulting ligand structures were geometrically optimized using OpenBabel (v3.0.0) [40] with the GAFF force field [41] to refine all bond lengths and angles. The ligands were then parameterized using the Antechamber program in AmberTools23 with AM1-BCC charge calculations. Missing parameters were checked using parmchk2 in AmberTools23 [38].

#### 4.3.3. Solvation and System Assembly

All systems were solvated in a TIP3P water box [42] extending 10 Å beyond the solute in each direction. Na^+^ and Cl^−^ ions were added to achieve a final salt concentration of 0.15 M. Topology and coordinate files were generated using the tLEaP module of AmberTools23. The ff19SB protein force field [43], GAFF [41] for ligand parameters, and TIP3P for water were used in all simulations.

#### 4.3.4. Simulation Protocol

To investigate the enzyme–substrate specificity in both native and swapped combinations, MD simulations were performed for the following systems: PSY with GGPP, CrtM with FSPP, PSY with FSPP, and CrtM with GGPP. All simulations were conducted using AMBER23 [38]. Energy minimization was performed in three stages: water, side chains, and the full system. The water and side-chain stages each consisted of 5000 steps (2500 steps of steepest descent followed by 2500 steps of conjugate gradient minimization), while the full-system stage consisted of 10,000 steps (5000 steps of steepest descent followed by 5000 steps of conjugate gradient minimization). A non-bonded cutoff of 9.0 Å was applied.

After minimization, the systems were heated under NVT conditions and equilibrated at 300 K under NPT conditions. Bonds involving hydrogen atoms were constrained using the SHAKE algorithm. The systems were equilibrated through several short MD stages with weak positional restraints on the backbone until a stable density was achieved. Temperature was controlled using Langevin dynamics with a random seed (ig = −1), and pressure was regulated at 1 bar using a Monte Carlo barostat with isotropic position scaling. After equilibration, a 400 ns production run was performed for each system. Each system was simulated in three independent replicates, yielding a total of 1200 ns of simulation time per system. The final 200 ns of each replica was used for data analysis, including the convergence analysis.

### 4.4. Data Analysis

Basic MD metrics, including root mean square deviation (RMSD), root mean square fluctuation (RMSF), and principal component analysis (PCA), were analyzed using CPPTRAJ (v6.18.1) and the PyTraj (v2.0.2) Python library [44]. Enzyme–substrate interactions were characterized with ProLIF (v2.0.3) [45] and MDAnalysis (v2.10.0) Python libraries [46,47].

## 5. Conclusions

Our integrative computational analysis suggested that substrate specificity in the PSY/CrtB–CrtM enzyme family may arise from coordinated structural evolution at multiple levels rather than redesigning of the conserved catalytic core. The principal findings are as follows. (1) PSY is simulated to exhibit significantly broader conformational plasticity than CrtM, particularly in the GGPP-bound state, with ligand-specific flexibility signatures at structurally meaningful loop positions. This predicted dynamic behavior contrasts with the preorganized, conformationally restricted architecture of CrtM. (2) A phylogenetically conserved hydrophobic helix (Helix-13) is predicted to distinguish PSY from CrtM homologs at the protein surface, suggesting that PSY specialization extends beyond pocket-level changes to include adaptation to membrane-associated functions. Together, these computational insights suggest a structural and dynamic framework for the rational engineering of carotenoid synthases with altered substrate specificities and underscore the need for experimental structural characterization of PSY/CrtB enzymes.

## Data Availability

The original contributions of this study are included in the article and Supplementary Material, available at https://github.com/ade-wagimon2/Supplementary-MDPI-1/ (accessed on 21 January 2026). For further inquiries, please contact the corresponding authors.

## Supplementary Materials

The following supporting information can be downloaded at: https://www.mdpi.com/article/10.3390/molecules31121995/s1; Figure S1: Helix Interaction Dynamics. (A) Chord diagrams representing inter-helix contacts for CrtM structures: Holo (3W7F), Inhibitor-bound (2ZCQ), Apo (2ZCO), and Holo (5IYS). The thickness of chords represents contact density; the Apo form exhibits a notable reduction in connectivity involving Helix-2 compared to ligand-bound states. (B) Detailed contact matrices for the 2ZCQ structure highlighting specific stabilizing interactions: Helix-3(Q33)–Helix-15(E236), Helix-2(F26)–Helix-8(L145), and Helix-2(F22)–Helix-9(L164). These interactions lock the binding cavity in the active conformation.; Figure S2. Convergence Analysis. The plot indicates a plateau in the plot after window time 50 and 75; Figure S3. (A) Sequence alignment of CrtM (3W7F and 5IYS) and PSY (CrtB from Euglena gracilis without the irregular region sequence, accession No BAU24764.1). Residues corresponding to the ligand-binding site are highlighted, with catalytic residues shaded in gray and the hydrophobic site marked in black. (B) Structural presentation of the Catalytic Site (gray clouded area) and Hydrophobic Site (black clouded area). (C) The Binding cavity sequences similarity percentage.; Figure S4. The B-factor value visualization of the 3W7F structure shows that the thicker the ribbon, the higher the B-factor value; Figure S5. OpenFold prediction confidence metrics for PSY substrate complexes; Table S1. Simulation Statistics.

## Abbreviations

The following abbreviations are used in this manuscript:

AM1-BCC	Austin Model 1–Bond Charge Correction
CrtB	Bacterial phytoene synthase
CrtM	Dehydrosqualene synthase
DMAPP	Dimethylallyl diphosphate
FPP	Farnesyl diphosphate
FPPS	Farnesyl diphosphate synthase
FSPP	Farnesyl thiopyrophosphate
GAFF	General AMBER force field
GGPP	Geranylgeranyl diphosphate
HPLC	High-performance liquid chromatography
IPP	Isopentenyl diphosphate
MD	Molecular dynamics
MEP	Methylerythritol phosphate
MSA	Multiple sequence alignment
MVA	Mevalonate
NPT	Constant pressure and temperature
NVT	Constant volume and temperature
PCA	Principal component analysis
PDB	Protein Data Bank
PDE	Predicted Distance Error
pLDDT	Predicted local distance difference test
PSY	Phytoene synthase
Rg	Radius of gyration
RMSD	Root-mean-square deviation
RMSF	Root-mean-square fluctuation
