# Supplementary figures and images for: Comparative Structural Modeling Suggests Distinct Signatures of Conformational Plasticity and Surface Physicochemistry in Phytoene Synthase and Dehydrosqualene Synthase
Source: Molecules. 2026 Jun 7;31(12):1995. doi: 10.3390/molecules31121995 (PMC13304436; doi:10.3390/molecules31121995)

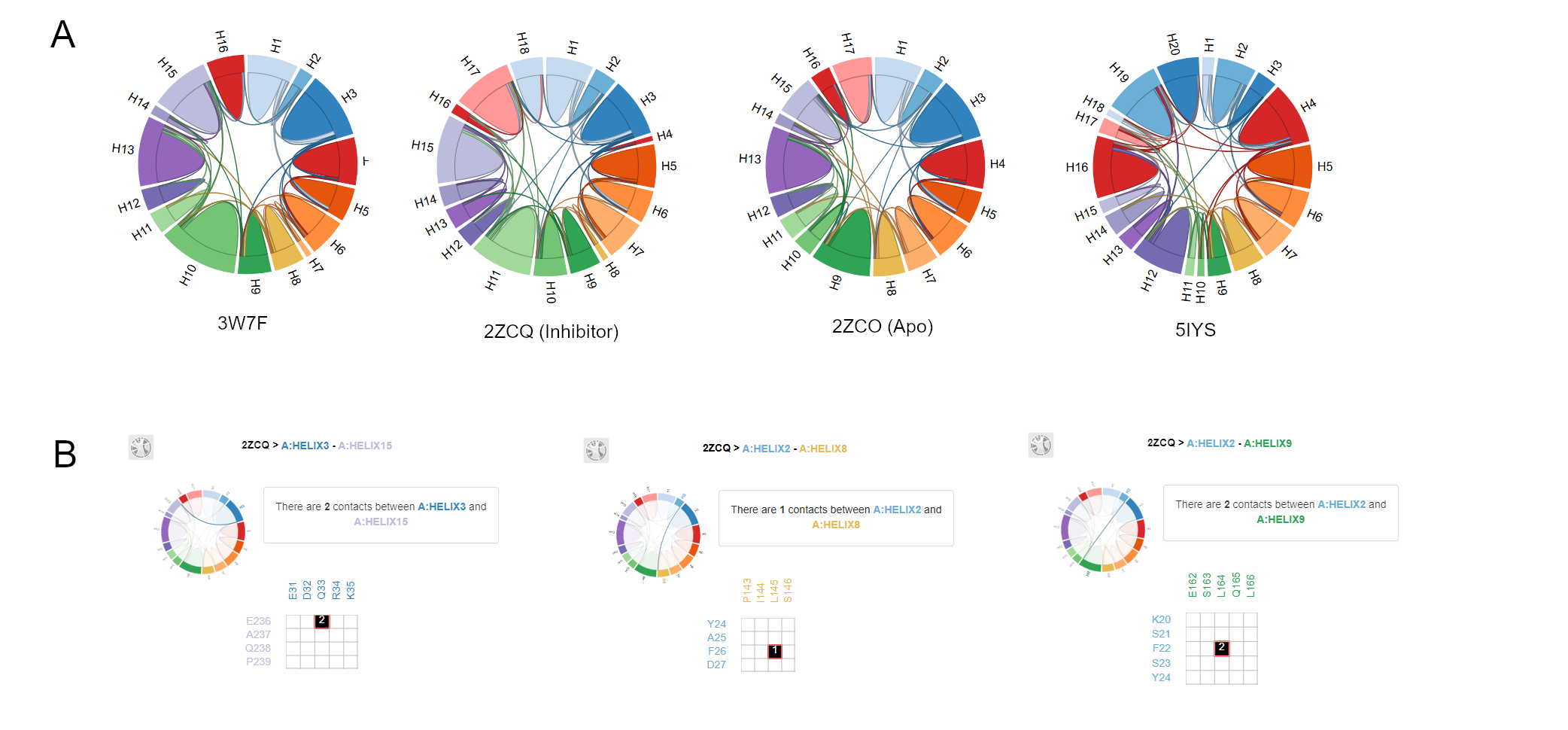

Supplement: Supplementary file 1 [file molecules-31-01995-s001.zip › Figure S1. Helices_Network.png]

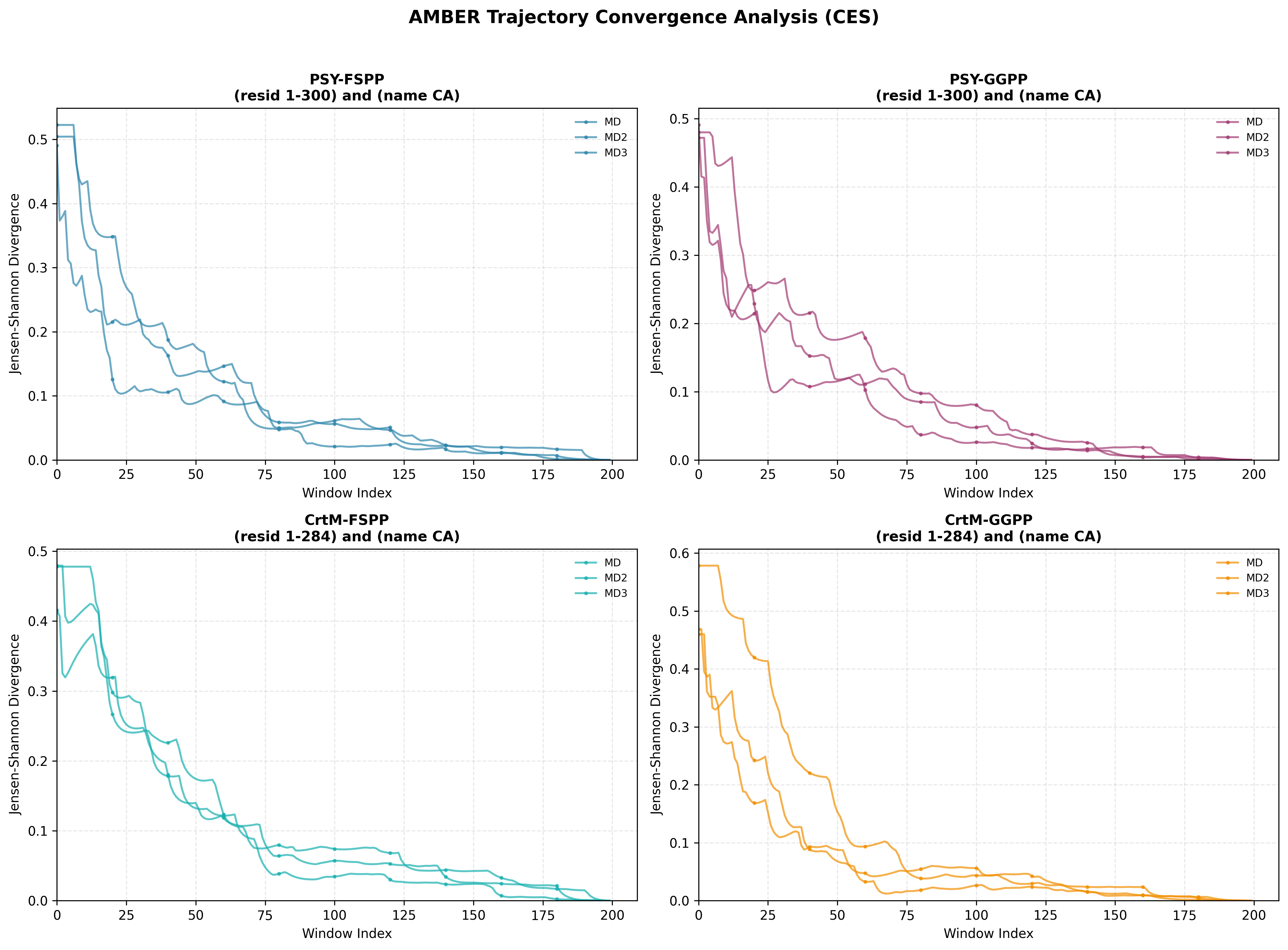

Supplement: Supplementary file 1 [file molecules-31-01995-s001.zip › Figure S2. amber_convergence_summary.png]

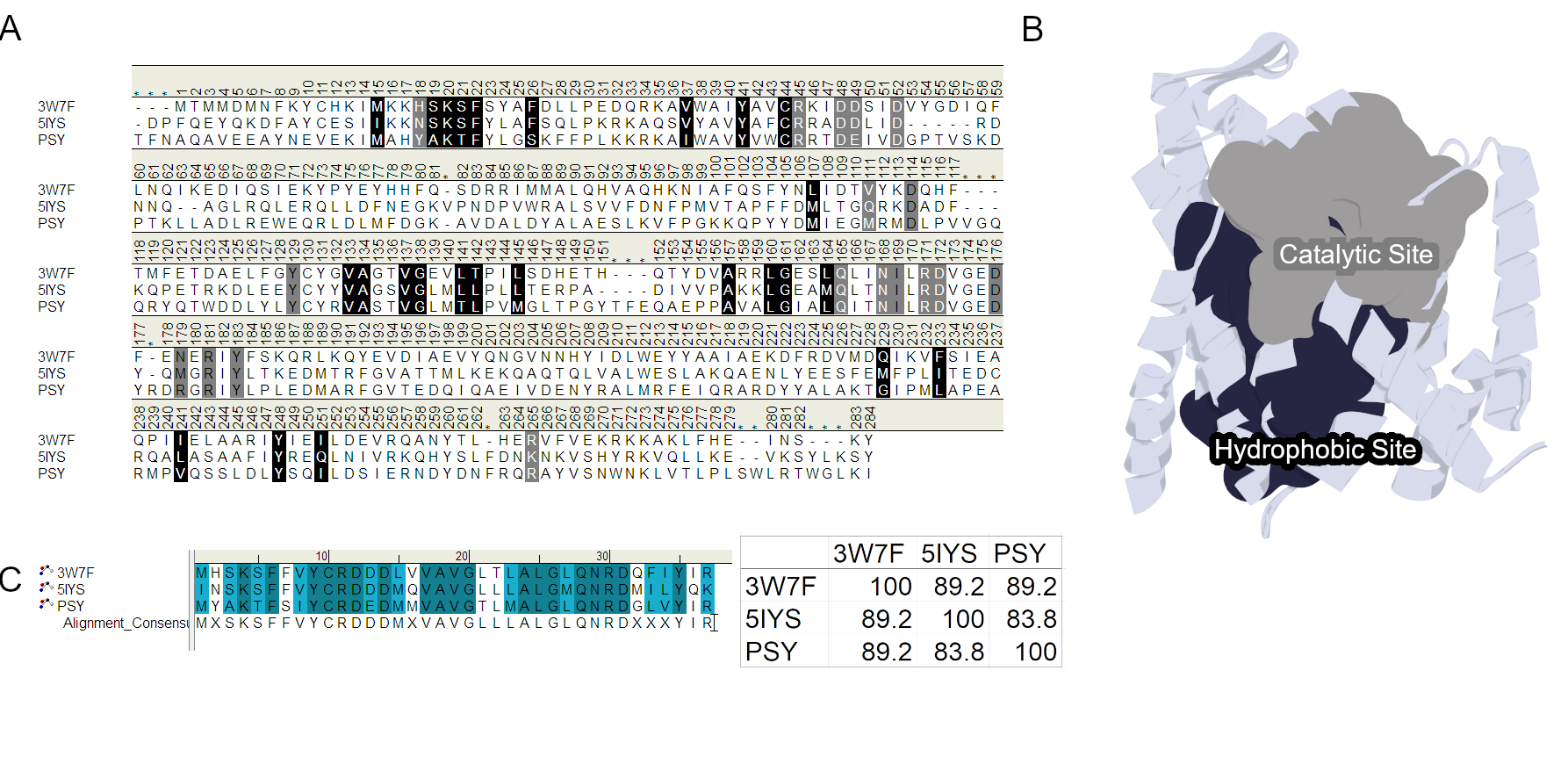

Supplement: Supplementary file 1 [file molecules-31-01995-s001.zip › Figure S3. msa_stucture.png]

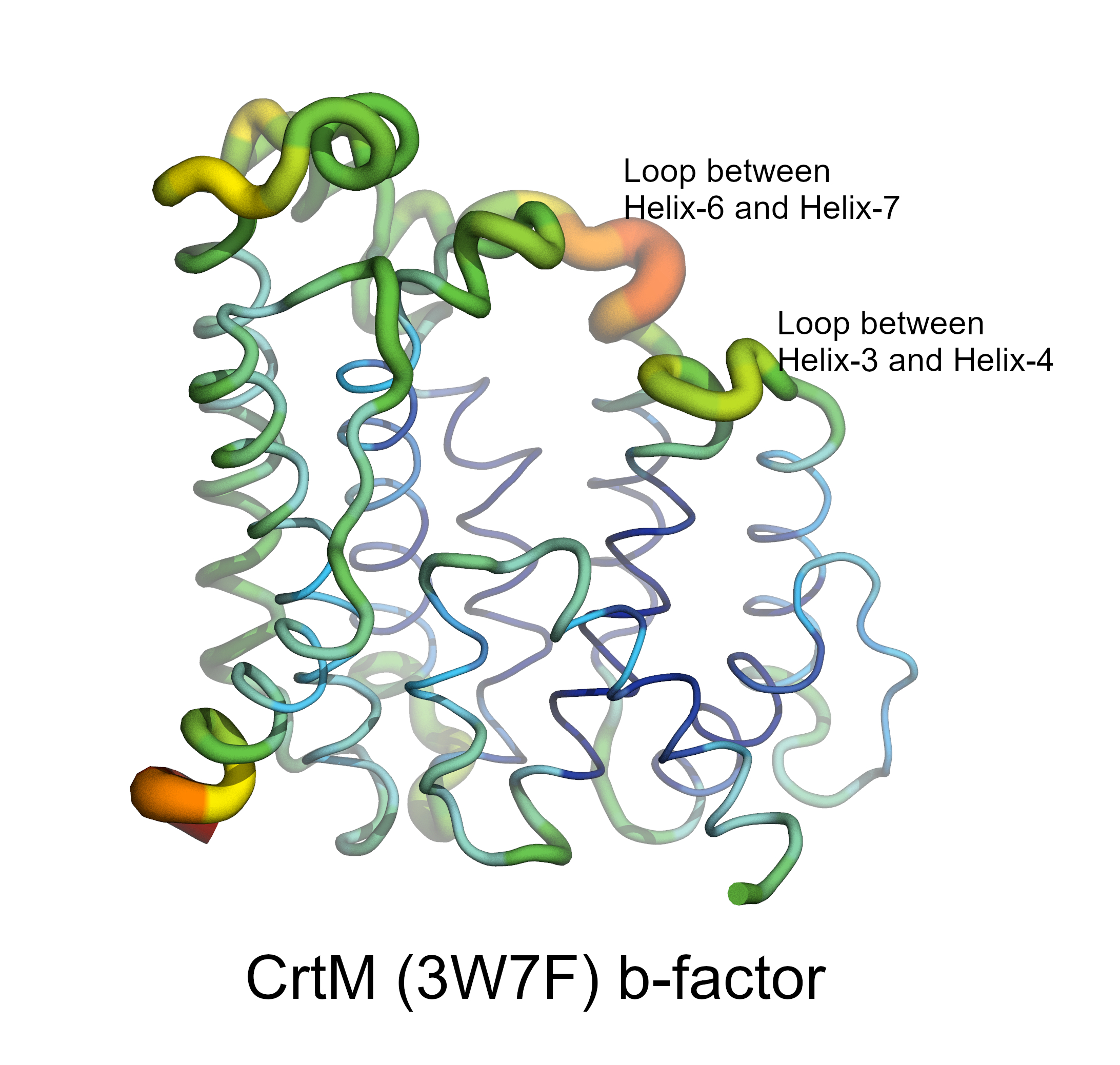

Supplement: Supplementary file 1 [file molecules-31-01995-s001.zip › Figure S4. bfactor.jpeg]

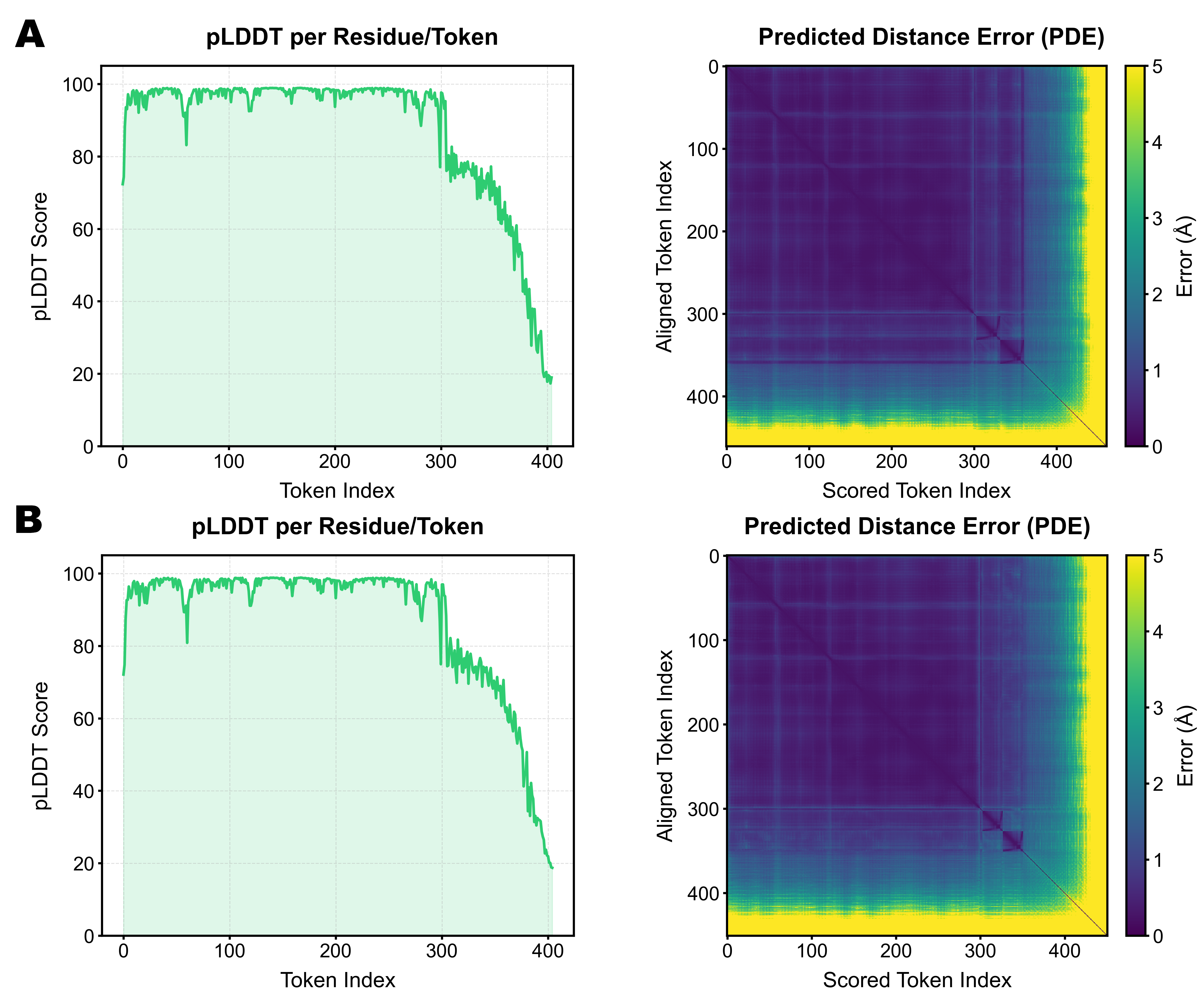

Supplement: Supplementary file 1 [file molecules-31-01995-s001.zip › Figure S5. confidence_visualization_model.png]
